# Regulation of the unfolded protein response via *S*-nitrosylation of sensors of endoplasmic reticulum stress

**DOI:** 10.1038/srep14812

**Published:** 2015-10-08

**Authors:** Ryosuke Nakato, Yu Ohkubo, Akari Konishi, Mari Shibata, Yuki Kaneko, Takao Iwawaki, Tomohiro Nakamura, Stuart A. Lipton, Takashi Uehara

**Affiliations:** 1 Department of Medicinal Pharmacology, Graduate School of Medicine, Dentistry, and Pharmaceutical Sciences, Okayama University, Okayama 700-8530, Japan; 2 Iwawaki laboratory, Education and Research Support Center, Graduate School of Medicine, Gunma University, Maebashi 371-8511, Japan; 3 Neuroscience and Aging Research Center, Sanford-Burnham-Prebys Medical Discovery Institute, La Jolla, California 92037, USA; 4 Department of Neurosciences, University of California, San Diego School of Medicine, La Jolla, California 92039, USA

## Abstract

Protein *S*-nitrosylation modulates important cellular processes, including neurotransmission, vasodilation, proliferation, and apoptosis in various cell types. We have previously reported that protein disulfide isomerase (PDI) is *S*-nitrosylated in brains of patients with sporadic neurodegenerative diseases. This modification inhibits PDI enzymatic activity and consequently leads to the accumulation of unfolded/misfolded proteins in the endoplasmic reticulum (ER) lumen. Here, we describe *S*-nitrosylation of additional ER pathways that affect the unfolded protein response (UPR) in cell-based models of Parkinson’s disease (PD). We demonstrate that nitric oxide (NO) can *S*-nitrosylate the ER stress sensors IRE1α and PERK. While *S*-nitrosylation of IRE1α inhibited its ribonuclease activity, *S*-nitrosylation of PERK activated its kinase activity and downstream phosphorylation/inactivation or eIF2α. Site-directed mutagenesis of IRE1α(Cys931) prevented *S*-nitrosylation and inhibition of its ribonuclease activity, indicating that Cys931 is the predominant site of *S*-nitrosylation. Importantly, cells overexpressing mutant IRE1α(C931S) were resistant to NO-induced damage. Our findings show that nitrosative stress leads to dysfunctional ER stress signaling, thus contributing to neuronal cell death.

The endoplasmic reticulum (ER) is involved in many essential cellular processes, including calcium homeostasis, steroid synthesis, protein folding and maturation, and quality control of newly synthesized proteins^1^,^2^. Chaperones in the ER facilitate protein folding and prevent accumulation of misfolded proteins. Correct folding of newly synthesized proteins that contain disulfide bonds and/or an *N*-glycan is facilitated by several ER chaperones, including protein disulphide isomerase (PDI), calnexin, and BiP^3^,^4^. Only correctly-folded proteins are transported from the rough ER to the Golgi complex for export. Severe cellular stress, engendered by hypoxia, energy deprivation, or exposure to excessive reactive oxygen or nitrogen species (including nitric oxide (NO)), can lead to ER stress. Such stress triggers the unfolded protein response (UPR)^5^,^6^,^7^,^8^, which, if terminated within a moderate amount of time, can lead to cytoprotection. However, prolonged ER stress upregulates the pro-apoptotic transcription factor C/EBP homologous protein (CHOP), leading to apoptotic cell death^9^,^10^.

The UPR is a stress response that prevents accumulation of unfolded proteins in the ER lumen. Unfolded proteins in the ER are detected by transmembrane ER stress sensors^11^,^12^. The three major ER stress-sensing proteins are PKR-like ER kinase (PERK), inositol-requiring enzyme 1 (IRE1), and activating transcription factor 6 (ATF6)^13^,^14^,^15^. These sensors transmit signals from the ER to the cytoplasm or nucleus, and activate the following three pathways: (i) suppression of protein translation to halt the production of more unfolded proteins, (ii) induction of genes encoding ER molecular chaperones to facilitate protein folding, and (iii) activation of ER-associated degradation (ERAD) to decrease the accumulation of unfolded proteins in the ER^16^.

NO regulates numerous cell responses associated with proliferation, neurotransmission, synaptic plasticity, or cytotoxicity, in part via protein *S*-nitrosylation. This redox-mediated chemical modification occurs via oxidative reaction between NO and cysteine (Cys) thiol (or more properly thiolate anion) in the presence of an electron acceptor; alternatively, the reaction can proceed via transnitrosylation from one *S*-nitrosothiol to another^17^,^18^,^19^. We previously demonstrated that PDI is a target of NO under neurodegenerative conditions. *S*-Nitrosylation of PDI (forming SNO-PDI) inhibits its enzymatic activity and induces ER stress^20^. Additionally, we found that NO suppresses expression of mRNAs encoding ER stress markers. Therefore, we postulated that unidentified SNO*-*proteins might be involved in the UPR. In the present study, we discovered that a number of ER stress-sensing proteins are *S*-nitrosylated, suggesting a novel regulatory mechanism for UPR activation.

## Results

### NO regulates the IRE1α pathway during the UPR

**Figure 1:**
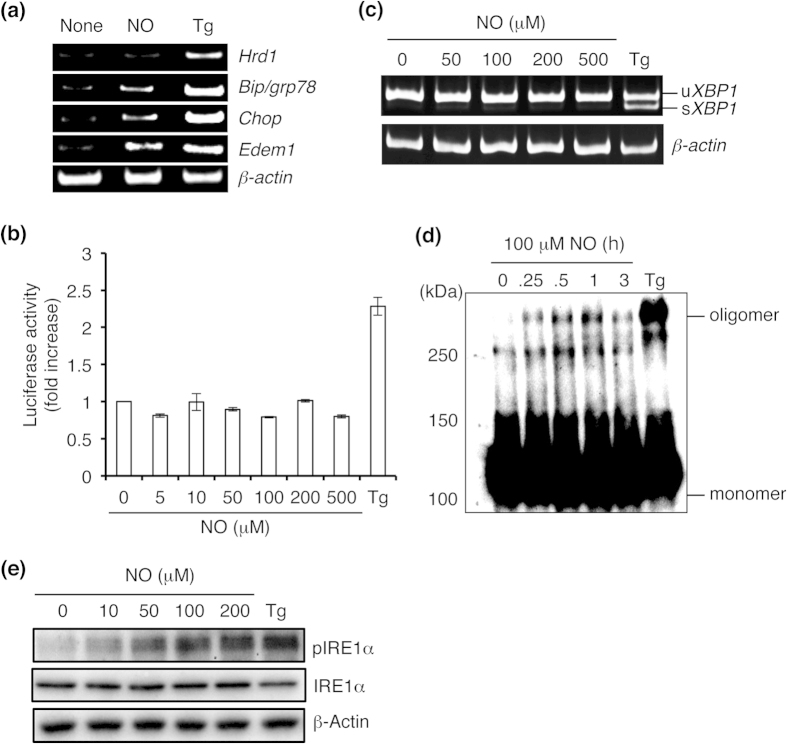
NO regulates the IRE1α-XBP1 pathway. (**a**) NO-stimulates ER stress-related gene expression. SH-SY5Y cells were exposed to 100 μm GSNO (NO) or 1 μM thapsigargin (Tg) for 12 h. RT-PCR was performed using specific primers for each mRNA. (**b**) NO does not activate *XBP1* mRNA signaling. SH-SY5Y cells were transfected with a vector expressing the XBP1–Luc reporter. After 48 h, the transfected cells were exposed to various concentrations of NO or 1 μM Tg, and luciferase assay was performed. Values expressed as mean ± s.e.m. (*n* = 5). (**c**) ER stress evoked by NO is independent of *XBP1* mRNA splicing. SH-SY5Y cells were treated with the indicated concentrations of NO for 12 h, and *XBP1* mRNA splicing was determined by performing RT-PCR. (**d**,**e**) NO induces oligomerization and phosphorylation of IRE1α. Lysates of SH-SY5Y cells exposed to NO for the indicated period of time were electrophoresed by performing native PAGE and then immunoblotted with anti-IRE1α antibody (**d**). After exposure to varying concentrations of NO for 30 min, the level of phosphorylated IRE1α was detected by western blotting (**e**).

We examined mRNA expression of specific ER stress markers in response to NO. Exposure to *S*-nitrosoglutathione (GSNO), a physiological NO donor, elevated the mRNA expression level of genes encoding BiP, CHOP, and EDEM1, but not HRD1 (Fig. 1a). Because HRD1 mRNA expression is known to be dependent on XBP1, it is possible that NO regulates this branch of the UPR via IRE1α^21^. To determine if NO modulates the UPR in this manner, we transfected SH-SY5Y neural cells with a vector expressing the fusion gene XBP1-luciferase (Luc) in order to monitor IRE1α activity^22^. Cells transfected with the XBP1–Luc expression vector showed detectable luminescence when treated with thapsigargin (Fig. 1b). However, exposure to high concentrations of NO did not significantly affect XBP1–Luc reporter activity, consistent with the notion that the IRE1α-XBP1 branch was regulated by NO. In addition, NO did not induce cytosolic splicing of *XBP1* mRNA (Fig. 1c). Next, we asked if NO impaired oligomerization or kinase activity of IRE1α. Empirically, we observed that exposure to NO did not attenuate oligomerization or phosphorylation of IRE1α (Fig. 1d,e).

### Neurotoxin 1-methyl-4-phenylpyridinium (MPP^+^) and ER stress attenuate the IRE1α pathway in an NO synthase (NOS)-dependent manner

**Figure 2:**
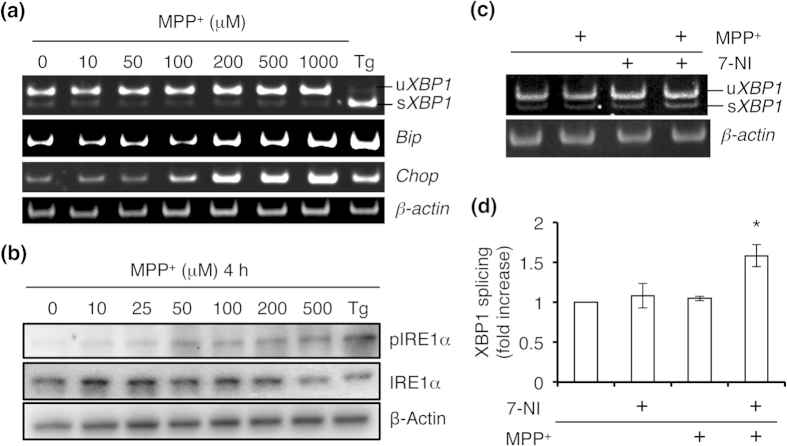
NO modulates MPP^+^-induced attenuation of the IRE1α pathway. (**a**) MPP^+^-stimulated ER stress-related gene expression. SH-SY5Y cells were incubated in MPP^+^ for 24 h. RT-PCR was performed using specific primers for each mRNA. (**b**) Cells were exposed to varying concentrations of MPP^+^ for 2 h, and western blotting was performed using anti-phospho-IRE1α antibody. (**c**) Cells were preincubated with 200 μM 7-NI for 6 h and then stimulated with 500 μM MPP^+^ for 12 h. Splicing of *XBP1* mRNA was assessed by RT-PCR. (**d**) Quantification of *XBP1* mRNA splicing is shown in (**c**). Values are expressed as mean ± s.e.m. (*n* = 4; *P < 0.05 by ANOVA).

**Figure 3:**
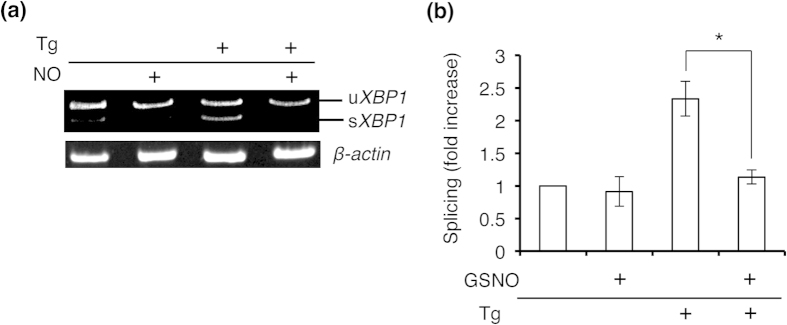
Prior exposure to NO inhibits ER stress-induced *XBP1* mRNA splicing. (**a**) NO attenuates Tg-stimulated *XBP1* mRNA splicing. Cells were preincubated with 100 μM NO for 30 min and then stimulated with 10 μM Tg for 2 h. Splicing of *XBP1* mRNA was assessed by performing RT-PCR. (**b**) Quantification of *XBP1* mRNA splicing is shown for data in (**a**). Values are expressed as mean ± s.e.m. (*n* = 4; *P* < 0.01 by ANOVA).

MPP^+^, which is known to cause a parkinsonian phenotype in rodents, non-human primates and humans, induces NO production and neuronal cell death, at least in part via ER stress^23^. Therefore, we tested whether MPP^+^ affected UPR signaling. We found that MPP^+^ induced mRNA expression of *BiP* and *CHOP* in a concentration-dependent manner; however, neither splicing of *XBP1* mRNA nor phosphorylation of IRE1α was detected under these conditions (Fig. 2a,b). To determine whether attenuation of *XBP1* mRNA splicing was mediated by NO, we treated SH-SY5Y cells with the NOS inhibitor 7-nitroindazole (7-NI), and found that splicing was significantly ameliorated (Fig. 2c,d). To further test the effects of ER stress and NO on IRE1α–XBP1 signaling, we induced ER stress with thapsigargin in the presence and absence of exogenous NO. Although thapsigargin clearly stimulated *XBP1* mRNA splicing, pre-incubation with NO completely abrogated this response (Fig. 3a,b). These findings are consistent with the notion that the ribonuclease activity of IRE1α is regulated by redox state, specifically via reaction with NO.

### *S*-Nitrosylation of IRE1α at Cys931 attenuates its ribonuclease activity

**Figure 4:**
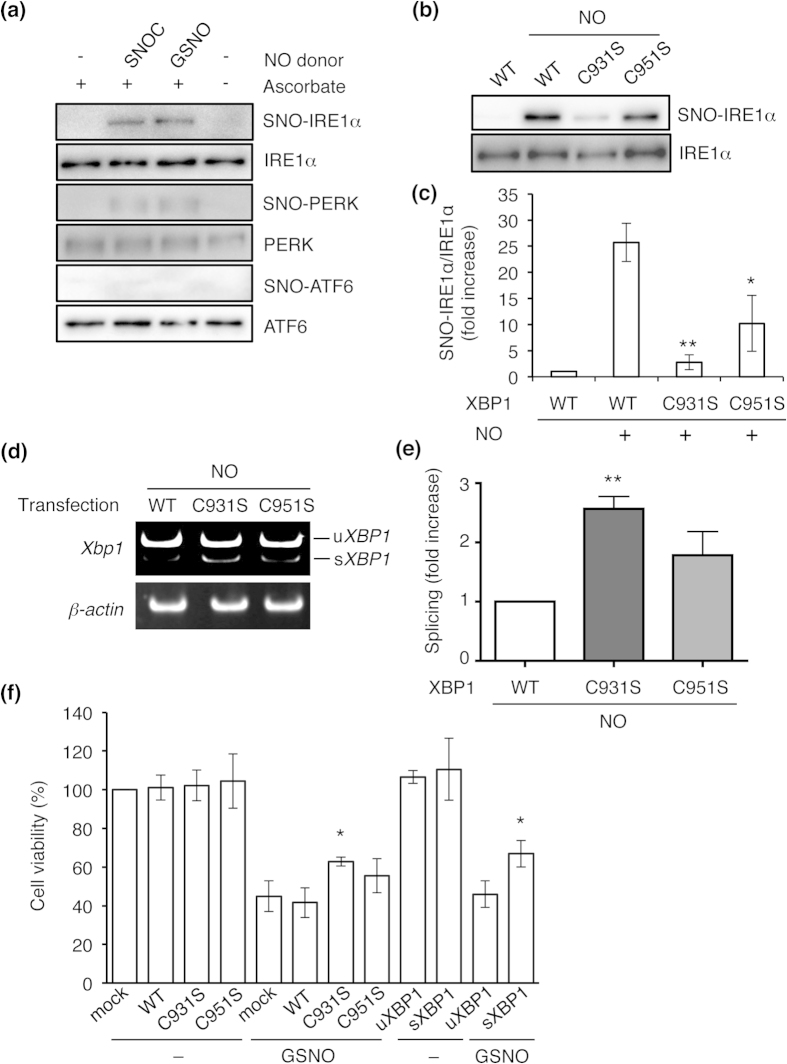
*S*-nitrosylation of ER stress sensor proteins. (**a**) SH-SY5Y neural cells transduced with vectors expressing wild-type IRE1α, PERK, or ATF6 were incubated with physiological NO donors (SNOC or GSNO) to determine *S*-nitrosylation. Control cells were exposed to old SNOC, from which NO had been dissipated, or glutathione. *S*-Nitrosylated proteins were detected by performing biotin-switch assays. (**b**) Top: SH-SY5Y neural cells, transduced with wild-type (WT) or C-to-S HA-tagged IRE1α mutants, were exposed to 100 μM SNOC or control for 1 h. SNO–IRE1α was detected by biotin-switch assay with anti-HA antibody. Bottom: Total IRE1α. (**c**) Quantification of *XBP1* mRNA splicing is shown from (**b**). Values are mean ± s.e.m. (*n* = 4; *P* < 0.05 or 0.01 by ANOVA). (**d**) *XBP1* mRNA splicing in IRE1α-null MEFs. IRE1α-null MEFs transduced with WT or IRE1α C-to-S mutants were exposed to 100 μM SNOC for 12 h, and *XBP1* mRNA splicing was assessed by performing RT-PCR. (**e**) Quantification of *XBP1* mRNA splicing. Values are mean ± s.e.m. (*n* = 4; *P* < 0.05 by ANOVA). (**f**) Overexpression of IRE1α(C931S) mutant or spliced *XBP1* mRNA prevented NO-induced cell death of IRE1α-null MEFs. Cell viability was estimated by performing WST-1 assay (see Methods). Values are mean ± s.e.m. (*n* = 4; *P* < 0.05 by ANOVA).

Next, we determined if NO could *S*-nitrosylate the UPR components IRE1α, PERK, or ATF6. By biotin-switch assay, we found that NO mediated *S*-nitrosylation of IRE1α (to form SNO-IRE1) and PERK (forming SNO-PERK), but not ATF6 (Fig. 4a). Therefore, we hypothesized that NO may modulate IRE1α endoribonuclease activity by *S*-nitrosylating the enzyme. Along these lines, two cysteine residues, at positions 931 and 951, are known to reside in the kinase-extension nuclease (KEN) domain of IRE1α^24^,^25^. To determine if these residues were the target site(s) of *S*-nitrosylation, we substituted each cysteine for serine and assessed SNO–IRE1α formation by performing biotin-switch assays. After 24 h, the cells were exposed to *S*-nitrosocysteine (SNOC) or control conditions and were monitored for SNO–IRE1α formation. We found that the IRE1α(C931S) mutant nearly totally abrogated *S*-nitrosylation while the C951S mutant had a more modest effect (Fig. 4b,c). These results are consistent with the notion that Cys-931 is the predominant nitrosylation site on IRE1α. To determine if this *S*-nitrosylation affected endonuclease activity, IRE1α–null mouse embryonic fibroblasts (MEFs) were used^26^. The MEFs were transfected with vectors expressing wild-type (WT) HA-tagged IRE1α or Cys mutant. NO-induced inhibition of IRE1α endonuclease activity was significantly ameliorated in MEFs expressing the IRE1α (C931S) mutant (Fig. 4d,e). To our knowledge, this is the first demonstration that Cys931 is involved in allosteric regulation of IRE1α activity.

Next, to test the potential role of *S*-nitrosylation of IRE1α in cell death, we investigated the effects of the Cys mutants on NO-induced cell damage in IRE1α-null MEFs. Transient expression of IRE1α (C931S) significantly restored cell viability compared with mock transfectants (Fig. 4f). Additionally, as expected, overexpression of spliced *XBP1* mRNA, but not unspliced *XBP1* mRNA, attenuated NO-induced cell death (Fig. 4f).

### NO enhances eIF2α phosphorylation

**Figure 5:**
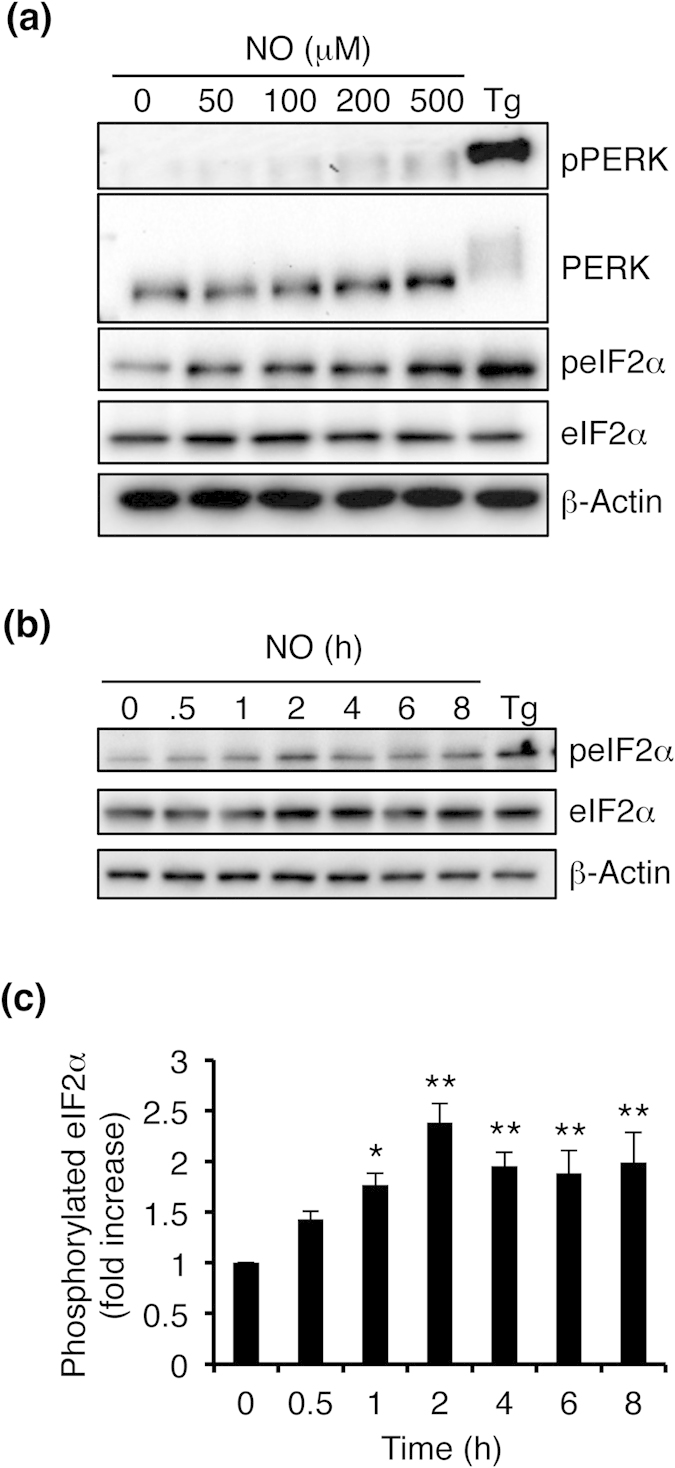
NO-induced phosphorylation of eIF2α. (**a**) Effect of NO treatment on the phosphorylation of PERK and eIF2α. Cells were exposed to varying concentrations of NO for 30 min, and levels of phosphorylated PERK, total PERK, phosphorylated eIF2α, total eIF2α, and β-actin were detected by immunoblotting. (**b**) Time-dependent eIF2α phosphorylation after exposure to 100 μM NO. (**c**) Quantification of eIF2α phosphorylation is shown for (**b**). Values are mean ± s.e.m. (*n* = 4; *P* < 0.05 or 0.01 by ANOVA).

As shown above, SNOC or GSNO can *S*-nitrosylate PERK, but not ATF6 (Fig. 4a). Therefore, we investigated whether NO influences PERK activity/phosphorylation in SH-SY5Y neural cells. ER stress is known to result in the oligomerization and autophosphorylation of PERK, which then phosphorylates and thus inactivates eIF2α, although other kinases can also influence eIF2α activity^27^. Empirically, we found that NO induced a modest but detectable degree of phosphorylation of PERK (Fig. 5a). Additionally, NO significantly enhanced phosphorylation of eIF2α in a concentration-dependent manner (Fig. 5a). This resulting phosphorylation was transient and reached a peak level ~2 h after stimulation (Fig. 5b,c).

## Discussion

The present study elucidated the effects of NO on major signaling pathways involved in the UPR. Previous reports had shown that NO reacts with PDI to form SNO–PDI in the ER lumen of human brains obtained from patients with several neurodegenerative diseases characterized by abnormal protein accumulation, including Parkinson’s disease (PD)^20^. Cell-based models of neurodegeneration produced by exposure to NO or mitochondrial toxins known to induce Parkinsonism also displayed formation of SNO–PDI. *S*-Nitrosylation of PDI was shown to impair protein folding, thus promoting ER stress and the UPR, as observed in PD^28^,^29^,^30^. Here, we found that NO also reacts with the major ER stress sensors, IRE1α and PERK, thus attenuating UPR and contributing to cell death.

Initially, we investigated whether the IRE1α-XBP1 pathway was affected by exposure to NO. We found that NO inhibited *XBP1* mRNA splicing, but did not affect oligomerization or phosphorylation of IRE1α. Additionally, we found that pre-incubation with NO strongly inhibited *XBP1* mRNA splicing induced by the well-known ER stress agent, thapsigargin. Moreover, our results show that exposure of SH-SY5Y neural cells to the PD-inducing agent MPP^+^, which generates NO, attenuated *XBP1* mRNA splicing in an NOS inhibitor-dependent manner. From our findings, we concluded that NO inhibited the endonuclease activity of IRE1α but not the kinase activity of its cytosolic domain, and that IRE1α did not lose its ability to sense the accumulation of unfolded proteins in the ER lumen after exposure to NO.

Next, we determined the Cys residue of IRE1α targeted by NO. Analysis of the atomic structure of the cytosolic domain of IRE1α showed that Cys931 and Cys951 are both located in the KEN domain^24^,^25^. Biotin-switch assay revealed that Cys931 is the predominant site of *S*-nitrosylation. Importantly, as evidence for a causal relationship of the effect of *S*-nitrosylation, transduction of IRE1α-null MEFs with the C931S mutant increased IRE1α endoribonuclease activity and protected cells from NO-induced cell death. These results indicate that the Cys931 residue of IRE1α is critical for redox signaling in the UPR. Such redox regulation of IRE1α activity has not been previously reported. We then tested whether NO can modulate other sensors of ER stress and observed *S*-nitrosylation of PERK but not ATF6.

Collectively, we found that NO can *S*-nitrosylate IRE1α and PERK to regulate the UPR. Because the IRE1α–XBP1 pathway functions prominently as an anti-apoptotic pathway, this redox-mediated inhibition by NO hinders cell survival during ER stress. In contrast, prolonged activation of the PERK–eIF2α–ATF4/ATF6 pathways is known to induce cell death via induction of CHOP. Thus, in conjunction with its inhibition of the IRE1α branch of the UPR, NO activation of the PERK-eIF2α pathway would further sensitize neural cells to apoptosis. Taken together, we have elucidated the effects of *S*-nitrosylation on ER stress sensors that mediate the UPR, and thus contribute to cell death pathways. These findings have important implications for the development new therapeutic approaches for PD and other neurodegenerative diseases associated with nitrosative stress and abnormal protein accumulation.

## Methods

### Materials

Biotin-HPDP was purchased from Pierce Chemical Co. Thapsigargin was obtained from Wako Pure Chemical Ltd. All other reagents were obtained from Sigma-Aldrich. Antibodies against IRE1α and phospho-IRE1α, PERK, phospho-PERK, ATF6, eIF2α, phospho-eIF2α, and β-actin were purchased from Cell Signaling Technology.

### Cell culture

Human SH-SY5Y cells and MEFs were maintained in Dulbecco’s modified Eagle’s medium supplemented with 10%(v/v) heat-inactivated fetal calf serum at 37 °C in humidified atmosphere of 5% CO_2_/95% air.

### Mutagenesis

Mutants of IRE1α were generated by substituting Ser for Cys residues using the QuickChange Site-Directed Mutagenesis Kit (Stratagene) according to the manufacturer’s instructions.

### Luciferase assay

The XBP1–Luc reporter (ERAI system) and the phRL–TK (Promega, Madison, WI) were used in the dual luciferase assay. SH-SY5Y cells were seeded in 24-well plates and were then transfected with the plasmids. The cells were lysed after 24 h of transfection, and luciferase assay was performed. Reporter activity was measured using Dual-Luciferase 1000 Assay System kit (Promega) and a luminometer (Berthold, Bad Wildbad, Germany). The results are expressed as mean ± s.e.m. of three experiments. Each value is normalized to fold induction in mock-transfected cells.

### RNA extraction and RT-PCR

Total RNA of cells was extracted using TRI reagent (Sigma-Aldrich). Transcriptor High Fidelity cDNA Synthesis Kit (Roche, Basel, Switzerland) was used to synthesize the cDNA according to the manufacturer’s instructions. The cDNA was amplified by using 24 cycles of PCR. The following primers were used: human *XBP1* sense primer, 5′-TTA CGA GAG AAA ACT CAT GGC C-3′; human *XBP1* antisense primer, 5′-GGG TCC AAG TTG TCC AGA ATG C-3′; human *EDEM1* sense primer, 5′-TCC ATA TCC TCG GGT GAA TC-3′; human *EDEM1* antisense primer, 5′-AAA TTC CAC CAG GAG GGA AC-3′; human *GRP78/BiP* sense primer, 5′-GTT TGC TGA GGA AGA CAA AAA GCT C-3′; human *GRP78/BiP* antisense primer 5′-CAC TTC CAT AGA GTT TGC TGA TAA TTG-3′; human *HRD1* sense primer 5′-GCA CAC CTT CCC ACT CTT TG-3′; human *HRD1* antisense primer 5′-TGG CAC CAG TCA CCA TCT CT-3′; human *CHOP* sense primer, 5′-CTC TGG CTT GGC TGA CTG A-3′; human *CHOP* antisense primer, 5′-CTT CAG CTA GCT TGT CCA CT-3′; human β*-actin* sense primer, 5′-CCT GAC GGC CAG GTC ATC-3′; human β*-actin* antisense primer, 5′-GGA CTC GTC ATA CTC CTG-′’. PCR products were analyzed by performing agarose gel electrophoresis on a 1.5% gel.

### Western blotting analysis

SH-SY5Y cells were treated as indicated above, harvested, washed with PBS, and lysed in ice-cold lysis buffer (50 mM Tris–HCl pH 7.5, 150 mM NaCl, 1 mM EDTA, and 1% NP-40 containing a protease inhibitor cocktail) for 10 min. For detecting of ATF6, the cells were preincubated with or without 50 μM cycloheximide and 5 μM MG-132 for 3 h and were further incubated during NO treatment. After quantification with Bradford assay, the proteins were boiled in sample loading buffer for 5 min and were electrophoresed by performing SDS-PAGE. The proteins (10 μg/lane) were then transferred onto a PVDF membrane. The membrane was blocked with 5% BSA or non-fat dry milk in Tris-buffered saline containing 0.1% Tween-20 for 1 h at room temperature. The membrane was then incubated overnight at 4 °C with the antibodies indicated above. The proteins were detected using Western Lightning Ultra ECL-HRP substrate (PerkinElmer, Waltham, MA) and visualized using the ChemiDoc MP Imaging System (Bio-Rad).

### Biotin-switch assay for *S*-nitrosylated proteins (SNO-P)

Cell lysates were prepared in HENT buffer (250 mM Hepes, pH7.5, 1 mM EDTA, 0.1 mM neocuproine, 0.1% SDS, and 1% Triton X-100). Protein concentration ranges were tested using the biotin-switch assay. Typically, 0.8 mg of cell lysate and up to 1.6 mg of tissue extract were used. Samples were mixed with blocking buffer (2.5% SDS and 20 mM methylmethanethiosulfonate (MMTS) in HEN buffer) and were incubated at 50 °C for 30 min to block free thiol groups. After removing excess MMTS by acetone precipitation, nitrosothiols were then reduced to free thiols by using 1 mM ascorbate. The newly formed thiols were linked to the sulfhydryl-specific biotinylating reagent biotin–HPDP. The biotinylated proteins were pilled down using streptavidin–agarose beads, and SNO-P remaining in the samples was detected by performing western blotting.

### Assessment of cell viability

Cell viability was measured in triplicate in 24-well plates by performing a quantitative colorimetric assay with 5-(2, 4-bis[sodiooxysulfonyl]phenyl)-2-(4-nitrophenyl)-3-(4-iodophenyl)-2H-tetrazole-3- ium (WST-1). Briefly, IRE1α-null MEFs were transfected with 0.4 μg of each gene and were incubated for 24 h. Next, the cells were treated with 100 μM GSNO for 24 h. Cell viability was expressed as the ration of signal obtained from treated cells and signal obtained from control cells multiplied by 100 (% control).

### Statistical analysis

All experiments were independently performed at least three times. All data are expressed as the mean ± s.e.m. Statistical comparisons were performed using an ANOVA with a Bonferroni correction conducted post hoc using Graphpad Prism 5 (Graphpad Software, La Jolla, CA, USA). *P* values < 0.05 were considered to be significant.

## Additional Information

**How to cite this article**: Nakato, R. *et al.* Regulation of the unfolded protein response via *S*-nitrosylation of sensors of endoplasmic reticulum stress. *Sci. Rep.* **5**, 14812; doi: 10.1038/srep14812 (2015).

## References

[b1] Gething M. J. & Sambrook J. Protein folding in the cell. Nature 355, 33–45 (1992). 1731198 10.1038/355033a0

[b2] Ellgaard L., Molinari M. & Helenius A. Setting the standards: quality control in the secretory pathway. Science 286, 1882–1888 (1999). 10583943 10.1126/science.286.5446.1882

[b3] Rapoport T. A. Transport of proteins across the endoplasmic reticulum membrane. Science 258, 931–936 (1992). 1332192 10.1126/science.1332192

[b4] Kaufman R. J. Orchestrating the unfolded protein response in health and disease. Clin Invest. 110, 1389–1398 (2002). 10.1172/JCI16886 PMC151822 12438434

[b5] Malhotra J. D. & Kaufman R. J. Endoplasmic reticulum stress and oxidative stress: a vicious cycle or a double-edged sword? Antioxid Redox Signal. 9, 2277–2793 (2007). 17979528 10.1089/ars.2007.1782

[b6] Helenius A., Marquardt T. & Braakman I. The endoplasmic reticulum as a protein-folding compartment. Trends Cell Biol. 2, 227–231 (1992). 14731479 10.1016/0962-8924(92)90309-b

[b7] Schröder M. & Kaufman R. J. ER stress and the unfolded protein response. Mutat Res. 569, 29–63 (2005). 15603751 10.1016/j.mrfmmm.2004.06.056

[b8] Ron D. & Walter P. Signal integration in the endoplasmic reticulum unfolded protein response. Nat Rev Mol Cell Biol. 8, 519–529 (2007). 17565364 10.1038/nrm2199

[b9] Friedman A. D. GADD153/CHOP, a DNA damage-inducible protein, reduced CAAT/enhancer binding protein activities and increased apoptosis in 32D c13 myeloid cells. Cancer Res. 56, 3250–3256 (1996). 8764117

[b10] Zinszner H. *et al.* CHOP is implicated in programmed cell death in response to impaired function of the endoplasmic reticulum. Genes Dev. 12, 982–995 (1998). 9531536 10.1101/gad.12.7.982 PMC316680

[b11] Kaufman R. J. Stress signaling from the lumen of the endoplasmic reticulum: coordination of gene transcription and translational controls. Genes Dev. 13, 1211–1233 (1999). 10346810 10.1101/gad.13.10.1211

[b12] Harding H. P. *et al.* Transcriptional and translational control in the mammalian unfolded protein response. Annu Rev Cell Dev Biol. 18, 575–599 (2002). 12142265 10.1146/annurev.cellbio.18.011402.160624

[b13] Mori K., Ma W., Gething M. J. & Sambrook J. A transmembrane protein with a cdc2^+^/CDC28-related kinase activity is required for signaling from the ER to the nucleus. Cell. 74, 743–756 (1993). 8358794 10.1016/0092-8674(93)90521-q

[b14] Harding H. P., Zhan Y. & Ron D. Protein translation and folding are coupled by an endoplasmic reticulum-resident kinase. Nature. 397, 271–274 (1999). 9930704 10.1038/16729

[b15] Yoshida H. *et al.* Identification of the cis-acting endoplasmic reticulum stress response element responsible for transcriptional induction of mammalian glucose-regulated proteins. Involvement of basic leucine zipper transcription factors. J Biol Chem. 273, 33741–33749 (1998). 9837962 10.1074/jbc.273.50.33741

[b16] Lee R. J. *et al.* Uncoupling retro-translocation and degradation in the ER-associated degradation of a soluble protein. EMBO J. 23, 2206–2215 (2004). 15152188 10.1038/sj.emboj.7600232 PMC419910

[b17] Liu L. *et al.* Essential roles of *S*-nitrosothiols in vascular homeostasis and endotoxic shock. Cell 116, 617–628 (2004). 14980227 10.1016/s0092-8674(04)00131-x

[b18] Foster M. W., Hess D. T. & Stamler J. S. Protein *S*-nitrosylation in health and disease: A current perspective. Trends Mol Med 15, 391–404 (2009). 19726230 10.1016/j.molmed.2009.06.007 PMC3106339

[b19] Foster M. W., Forrester M. T. & Stamler J. S. A protein microarray-based analysis of *S*-nitrosylation. Proc Natl Acad Sci USA 106, 18948–18953 (2009). 19864628 10.1073/pnas.0900729106 PMC2776442

[b20] Uehara T. *et al.* *S*-Nitrosylated protein-disulphide isomerase links protein misfolding to neurodegeneration. Nature 441, 513–517 (2006). 16724068 10.1038/nature04782

[b21] Shoulders M. D. *et al.* Stress-independent activation of XBP1s and/or ATF6 reveals three functionally diverse ER proteostasis environments. Cell Rep. 25, 1279–1292 (2013). 23583182 10.1016/j.celrep.2013.03.024 PMC3754422

[b22] Iwawaki T., Akai R., Kohno K. & Miura M. A transgenic mouse model for monitoring endoplasmic reticulum stress. Nat Med 10, 98–102 (2004). 14702639 10.1038/nm970

[b23] Przedborski S. *et al.* Role of neuronal nitric oxide in 1-methyl-4-phenyl-1,2,3,6-tetrahydropyridine (MPTP)-induced dopaminergic neurotoxicity. Proc Natl Acad Sci USA 93, 4565–4571 (1996). 8643444 10.1073/pnas.93.10.4565 PMC39317

[b24] Lee K. P. *et al.* Structure of the dual enzyme Ire1 reveals the basis for catalysis and regulation in nonconventional RNA splicing. Cell. 132, 89–100 (2008). 18191223 10.1016/j.cell.2007.10.057 PMC2276645

[b25] Ali M. M. *et al.* Structure of the Ire1 autophosphorylation complex and implications for the unfolded protein response. EMBO J. 30, 894–905 (2011). 21317875 10.1038/emboj.2011.18 PMC3049214

[b26] Oikawa D., Kimata Y., Kohno K. & Iwawaki T. Activation of mammalian IRE1alpha upon ER stress depends on dissociation of BiP rather than on direct interaction with unfolded proteins. Exp Cell Res. 315, 2496–2504 (2009). 19538957 10.1016/j.yexcr.2009.06.009

[b27] Cláudio N., Dalet A., Gatti E. & Pierre P. Mapping the crossroads of immune activation and cellular stress response pathways. EMBO J. 32, 1214–1224 (2013). 23584529 10.1038/emboj.2013.80 PMC3642686

[b28] Gasperini L. *et al.* Prion protein and copper cooperatively protect neurons by modulating NMDA receptor through S-nitrosylation. Antioxid Redox Signal. 22, 772–784 (2015). 25490055 10.1089/ars.2014.6032 PMC4361008

[b29] Nakamura T. *et al.* Aberrant protein S-nitrosylation contributes to the pathophysiology of neurodegenerative diseases. Neurobiol Dis. S0969–9961, 00089–3 (2015). 10.1016/j.nbd.2015.03.017 PMC4575233 25796565

[b30] Chung. K. K. Studying nitrosative stress in Parkinson’s disease. Methods Mol Biol. 1292, 195–201 (2015). 25804757 10.1007/978-1-4939-2522-3_14

